# Auditory Verbal Cues Alter the Perceived Flavor of Beverages and Ease of Swallowing: A Psychometric and Electrophysiological Analysis

**DOI:** 10.1155/2013/892030

**Published:** 2013-08-27

**Authors:** Aya Nakamura, Satoshi Imaizumi

**Affiliations:** Department of Communication Science and Disorders, Faculty of Health and Welfares, Prefectural University of Hiroshima, 1-1 Gakuen, Mihara, Hiroshima 723-0053, Japan

## Abstract

We investigated the possible effects of auditory verbal cues on flavor perception and swallow physiology for younger and elder participants. Apple juice, aojiru (grass) juice, and water were ingested with or without auditory verbal cues. Flavor perception and ease of swallowing were measured using a visual analog scale and swallow physiology by surface electromyography and cervical auscultation. The auditory verbal cues had significant positive effects on flavor and ease of swallowing as well as on swallow physiology. The taste score and the ease of swallowing score significantly increased when the participant's anticipation was primed by accurate auditory verbal cues. There was no significant effect of auditory verbal cues on distaste score. Regardless of age, the maximum suprahyoid muscle activity significantly decreased when a beverage was ingested without auditory verbal cues. The interval between the onset of swallowing sounds and the peak timing point of the infrahyoid muscle activity significantly shortened when the anticipation induced by the cue was contradicted in the elderly participant group. These results suggest that auditory verbal cues can improve the perceived flavor of beverages and swallow physiology.

## 1. Introduction

Pureed or minced food, which is served to patients suffering from dysphagia to prevent aspiration, is not easily recognized based on appearance. Individuals with disorders in the anticipatory stage may have difficulties in recognizing even ordinary foods. These difficulties in recognition may have a negative influence on flavor perception, resulting in decreased appetite. Previous reports, however, have suggested that nonverbal as well as verbal information can have significant positive effects on flavor perception. For example, the perceptual rating score of the flavor of fruit juice increased when pictures of juice were shown during ingestion [1]. Potato chips were perceived as being crisper and fresher when either the overall level or the level of the high-frequency components of biting sounds was amplified [2]. Swallowing behavior is initiated more quickly when drinking water while viewing photographs of food than photographs of common items [3, 4]. A significant positive effect of verbal priming on olfactory perception was also reported, that is, participants rated the affective value of a tested odor as being more pleasant when labeled “cheddar cheese” than when labeled “body odor" [5]. These reports suggest that nonverbal as well as verbal information could be utilized to improve flavor perception and to enhance appetite even for pureed or minced foods that have an unfamiliar appearance. Thus, the purpose of this study was to investigate whether spoken information about food before ingesting has a positive effect on flavor perception and swallowing physiology. If so, then this technique could facilitate dysphagia rehabilitation.

## 2. Materials and Methods

### 2.1. Participants

Participants were screened for any clinical signs of hearing disorders, dysgeusia, dysosmia, dysphagia, and for any medical problems or medications that might affect hearing, tasting, smelling, or swallowing. Each participant gave his/her informed consent prior to the study. Participants were asked to refrain from drinking and eating for at least 2 hours before the experimental session. Participants in Experiments 1 and 2 were recruited separately.

**Experiment 1**

Participants were 24 people (7 men and 17 women) between the ages of 20 and 69 years.

**Experiment 2**

Participants were divided into two groups based on age: one group of 11 younger people (1 man and 10 women) between the ages of 20 and 30 years (mean age of 21.7) and one group of 8 elder people (3 men and 5 women) between the ages of 65 and 75 years (mean age of 68.4) were included.

### 2.2. Stimulus

Five mL of apple juice, aojiru (grass juice), or water was placed on the dorsum of each participant's tongue by the examiner using a 10 mL syringe (SS-10ESZ30, NIPRO). All beverages were presented at room temperature (22-23°C). The syringe was hidden by plastic tape. The name of the beverage (auditory verbal cue): “Ringo” (apple juice), “Aojiru” (grass juice), “Omizu” (water) or silence was presented through a speaker (PM-1, Fostex).

We did not use primary taste solution, because individuals with dysphagia usually eat food of complex flavor rather than primary taste. Stimuli were selected on the grounds that these three types of beverages are clearly different in flavor. 

### 2.3. Experimental Conditions

There were 2 experimental conditions: the absence condition (3 beverages × 3 times = 9 trials) and the presence condition (3 beverages × 3 auditory verbal cues, once each = 9 trials). These conditions included Accurate auditory verbal cues (the spoken cue correctly identified the beverage), Inaccurate (the spoken cue did not correctly identify the beverage), and Absence (absence of spoken cues).

### 2.4. Configuration

**Figure 1:**
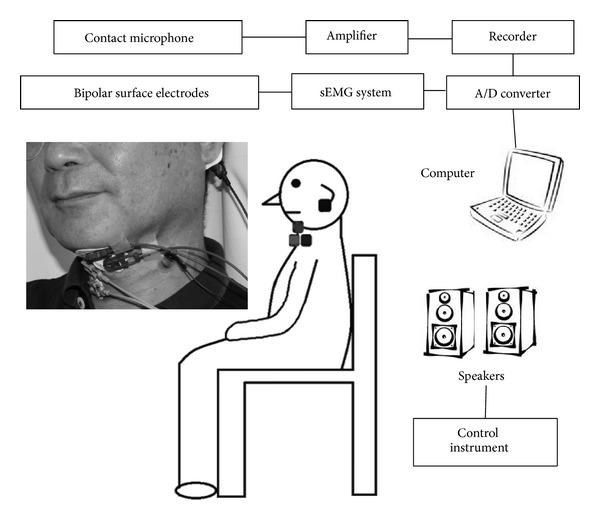
A schematic diagram of the instrumentation used for the collection of EMG data and swallowing sounds.

Surface electromyography (sEMG) and cervical auscultation were used. The configuration (Figure 1) included a sEMG system (Personal EMG 4CH, Oisakadenshikiki), A/D converter (ML870PowerLab8/30, AD Instruments), contact microphone (ECM-TL1, Sony), microphone amplifier (AT-MA2, Audio-Technica), recorder (CD-2, Roland), personal computer (VPCF12AHJ, Sony), control instrument (IS-703, IWATSU), and speaker (PM-1, Fostex).

The EMG activity associated with swallowing was recorded using bipolar surface electrodes (Arbo H24, Covidien Japan). All electrodes were taped to the skin using double-sided adhesive collars. Specific electrode positions were as follows: (a) a pair of bipolar surface electrodes was taped to the skin beneath the chin to record activity in the suprahyoid muscles, and (b) a pair of bipolar surface electrodes was placed on the lower right side of the thyroid cartilage to record activity in the infrahyoid muscles. A single electrode was affixed to the left side of the outer ear as ground.

Swallowing sounds were recorded using a contact microphone (ECM-TL1, Sony). The contact microphone was placed on the front of sternocleidomastoid muscle. The signals from the 2 pairs of electrodes and the contact microphone were recorded using an A/D converter (ML870PowerLab8/30, AD Instruments) with a sampling rate of 2 kHz. 

### 2.5. Experimental Design

**Figure 2:**
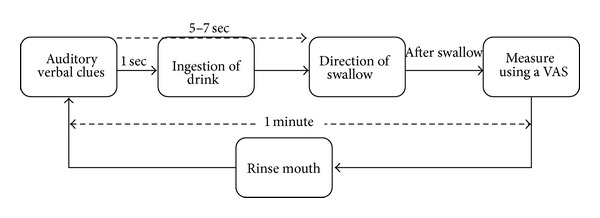
A schematic diagram of the time course of the task.

**Figure 3:**
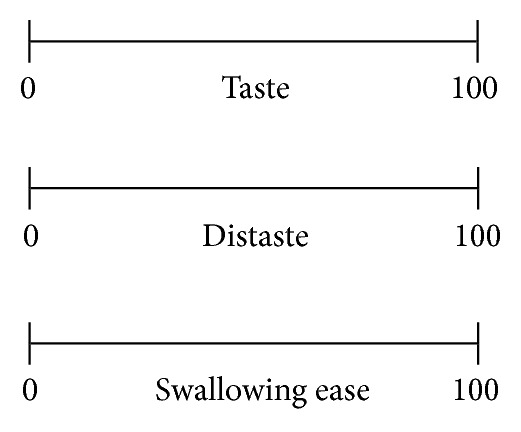
A visual analog scale used to measure the taste score, distaste score, and swallowing ease score.

The experiment was carried out as follows (Figure 2): during the study, participants sat in a chair. Participants were asked to imagine the flavor of a beverage based on auditory verbal cues (name of the beverage), and then a beverage was injected into their mouth. They were instructed to hold the beverage in their mouth for 5–7 s until given direction to swallow. They were also instructed to swallow the beverage in a single swallow. The participants rated flavor perception and ease of swallowing using a visual analog scale (VAS) (Figure 3). Participants rinsed their mouth with water after each swallow. Every participant completed the absence trials first, followed by the auditory verbal cue conditions. 

Beverages and auditory verbal cues were presented in a random order. 

### 2.6. Measurements

#### 2.6.1. VAS Scores

We used “taste score” as an indicator of delicious, tasty, nice flavor and “distaste score” as an indicator of no good, nasty, bad flavor. “Swallowing ease” was used as an indicator of ease of bolus transit pharynx.

We used both “taste score” and “distaste score” because we assumed that distaste score is not an indication in the opposite of the taste score's spectrum. Taste score is the indicator of the good or no taste when food intake is possible and safety. On the other hand, distaste score is the indicator of the taste, including the judgment of whether or not food intake is possible.

#### 2.6.2. Physiological Parameters

**Figure 4:**
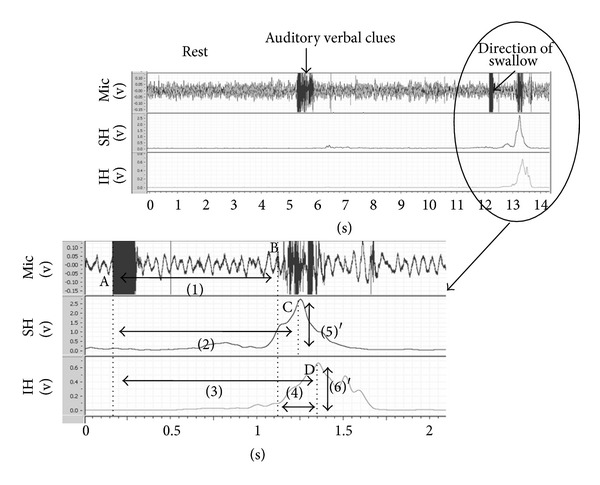
A schematic illustration of the definitions of the parameters for analysis. A: the point at which the direction to swallow is given, B: the point at which the swallowing sound starts, C: the point at the peak of suprahyoid muscle power, D: the point at the peak of infrahyoid muscle power. (1) The latency of the swallowing sound = B − A, (2) the latency of suprahyoid muscle activity = C − A, (3) the latency of infrahyoid muscle activity = D − A, (4) the interval between the onset of the swallowing sound and the peak timing of infrahyoid muscle activity = D − B, (5) the maximum value of suprahyoid muscle activity = (5)′, which is the peak of suprahyoid muscle power/the average suprahyoid muscle power at rest, and (6) the maximum value of infrahyoid muscle activity = (6)′, which is the peak of infrahyoid muscle power/the average infrahyoid muscle power at rest. SH: suprahyoid muscle power, IH: infrahyoid muscle power.

The parameters for analysis are shown in Figure 4. The original EMG signal was high-pass filtered (over 10 Hz), squared, and then low-pass filtered (under 10 Hz) to calculate the time series of the EMG power. We then divided the peak of the EMG power by the average power during the 5 sec. rest period before the first and final trials for each muscle to obtain the maximum values of suprahyoid muscle activity and infrahyoid muscle activity. The maximum value of muscle activity is the power value during swallowing when the power of the rest of the trial is equivalent to 1. Measurements for each swallow were conducted using the time series of the EMG power and Labchart 7 (AD Instruments). 

As swallowing parameters, we measured the latency of the swallowing sound, the latency of suprahyoid muscle activity, the latency of infrahyoid muscle activity, the interval between the onset of the swallowing sound and the peak timing point of infrahyoid muscle activity, the maximum value of suprahyoid muscle activity, and the maximum value of infrahyoid muscle activity. 

The latency of the swallowing sound was measured as the duration between A (the point at which direction to swallow was given) and B (the point at which the swallowing sound started). The latency of suprahyoid muscle activity was the duration between A and C (the peak point of suprahyoid muscle power). The latency of infrahyoid muscle activity was the duration between A and D (the peak point of infrahyoid muscle power). The interval between the onset of the swallowing sound and the peak timing point of infrahyoid muscle activity was the duration between B and D.

It has been noted that infrahyoid muscles are related to laryngeal decline [6]. The activation of the infrahyoid muscles occurs after the pharyngeal reflex starts, which implies that the activity takes place during the pharyngeal phase of swallowing. However, the latency of infrahyoid muscle activity (which is calculated by subtracting the onset of the direction to swallow from the peak of infrahyoid muscle EMG power) includes the duration of the oral phase of swallowing, which occurs before the pharyngeal reflex begins. The latency of the swallowing sound is reportedly affected by age [7–9]. Thus, the duration of the oral phase of swallowing is variable. Therefore, the duration of the oral phase (the latency of the swallowing sound) needs to be subtracted from the latency of the infrahyoid muscle activity for proper analysis of the laryngeal phase of swallowing. We calculated the interval between the onset of the swallowing sound and the peak timing point of infrahyoid muscle activity (the peak point of infrahyoid muscle EMG power minus the onset of the swallowing sound) to estimate the temporal duration from the onset of the pharyngeal reflex to the onset of laryngeal decline.

### 2.7. Statistical Analysis

#### 2.7.1. VAS Scores (Experiment 1)

Analyses of variances (ANOVA) were performed in Experiment 1 for flavor scores.

*Analysis 1*. To test the effects of the presence or absence of auditory verbal cues on flavor scores, we used a two-way ANOVA with Cue (presence, absence), Beverage (apple, water, aojiru) as independent factors. 

*Analysis 2*. To test the effects of accuracy of the auditory verbal cues on flavor scores, we used a two-way ANOVA with Accuracy (correct, incorrect), Beverage (apple, water, aojiru) as independent factors. Here, the “Correct” cue is a correct auditory verbal cue inducing appropriate anticipation for the beverage to be ingested, and the “Incorrect” cue is an incorrect auditory verbal cue with respect to the beverage to be ingested.

#### 2.7.2. Physiological Parameters (Experiment 2)

ANOVAs were performed in Experiment 2 for swallowing parameters. 

*Analysis 3*. To test the effects of the presence or absence of auditory verbal cues on swallowing parameters, we used a three-way ANOVA with Cue (presence, absence), Beverage (apple, water, aojiru), and Age (young, elderly) as independent factors. 

*Analysis 4*. To test the effects of accuracy of the auditory verbal cues on swallowing parameters, we used a three-way ANOVA with Accuracy (correct, incorrect), Beverage (apple, water, aojiru), and Age (young, elderly) as independent factors. 

*Analysis 5*. To clarify the interrelationship between the physiological parameters of swallowing, a repeated-measures ANOVA was applied to the latencies of swallowing sounds and the time points of maximum activity for each muscle, measured from the onset of the instruction to swallow. The interrelationships between the latencies were analyzed using a paired *t*-test. 

Statistical analysis was performed using Stat View 5.0 (Abacus Concepts, Cary, North Carolina, USA). Statistical significance was set at *P* < .05.

## 3. Results

### 3.1. VAS Scores (Experiment 1)

*Analysis 1*

**Figure 5:**
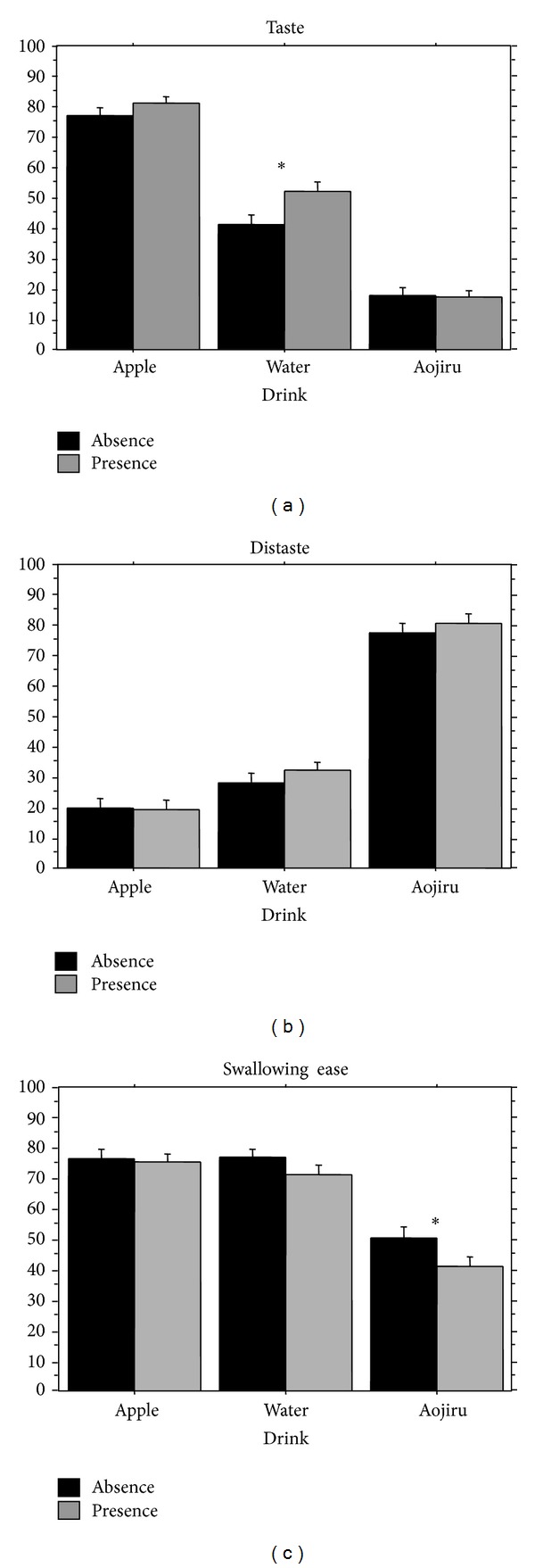
Comparisons of the taste score, the distaste score, and the swallowing ease score after swallowing with or without auditory verbal cues. Error bar: standard error.

*Taste Score*. In the analysis of the taste score, the ANOVA revealed significant main effects of Cue (*F*(1,426) = 4.9, *P* < .05) and Beverage (*F*(2,426) = 260, *P* < .01), as shown in Figure 5(a). The taste score significantly increased under the presence condition compared to the absence condition. The taste score significantly increased in the order of apple, water, and aojiru. 

*Distaste Score*. In the analysis of the distaste score, the ANOVA revealed significant main effect of Beverage (*F*(2,426) = 226, *P* < .01), as shown in Figure 5(b). The distaste score significantly increased in the order of aojiru, water, and apple. There was no significant effect of Cue on the distaste score.

*Swallowing Ease Score*. In the analysis of the swallowing ease score, the ANOVA revealed significant main effects of Cue (*F*(1,426) = 5.1, *P* < .05) and Beverage (*F*(2,426) = 63.2, *P* < .01), as shown in Figure 5(c). The swallowing ease score significantly decreased under the presence condition than the absence condition. The swallowing ease score significantly decreased for aojiru compared to apple and water.

*Analysis 2*

**Figure 6:**
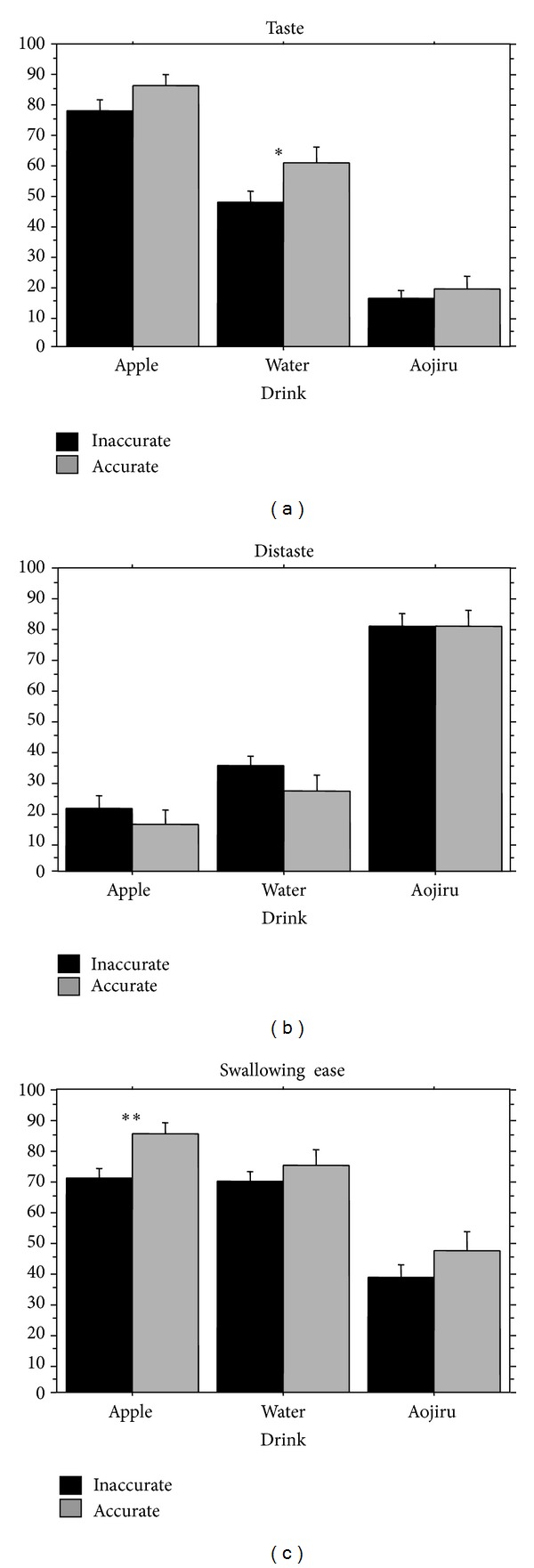
Comparisons of the taste score, the distaste score, and the swallowing ease score after swallowing when the participant's anticipation was primed by auditory verbal cues that were accurate versus inaccurate. Error bar: standard error.

*Taste Score*. In the analysis of the taste score, the ANOVA revealed significant main effects of Accuracy (*F*(1,210) = 6.5, *P* < .05) and Beverage (*F*(2,210) = 140.6, *P* < .01), as shown in Figure 6(a). The taste score significantly increased under the accuracy condition compared to the inaccuracy condition. The taste score significantly increased in the order of apple, water, and aojiru. 

*Distaste Score*. In the analysis of the distaste score, the ANOVA revealed significant main effect of Beverage (*F*(2,210) = 107.8, *P* < .01), as shown in Figure 6(b). The distaste score significantly increased in the order of aojiru, water, and apple. Accuracy did not have significant effects on the distaste score. 

*Swallowing Ease Score*. In the analysis of the swallowing ease score, the ANOVA revealed significant main effects of Accuracy (*F*(1,210) = 6.8, *P* < .01), and Beverage (*F*(2,210) = 35.7, *P* < .01) as shown in Figure 6(c). The swallowing ease score significantly decreased under the inaccurate condition compared to the accurate condition. The swallowing ease score of aojiru significantly decreased compared to apple and water. 

### 3.2. Physiological Parameters (Experiment 2)

*Analysis 3*

**Figure 7:**
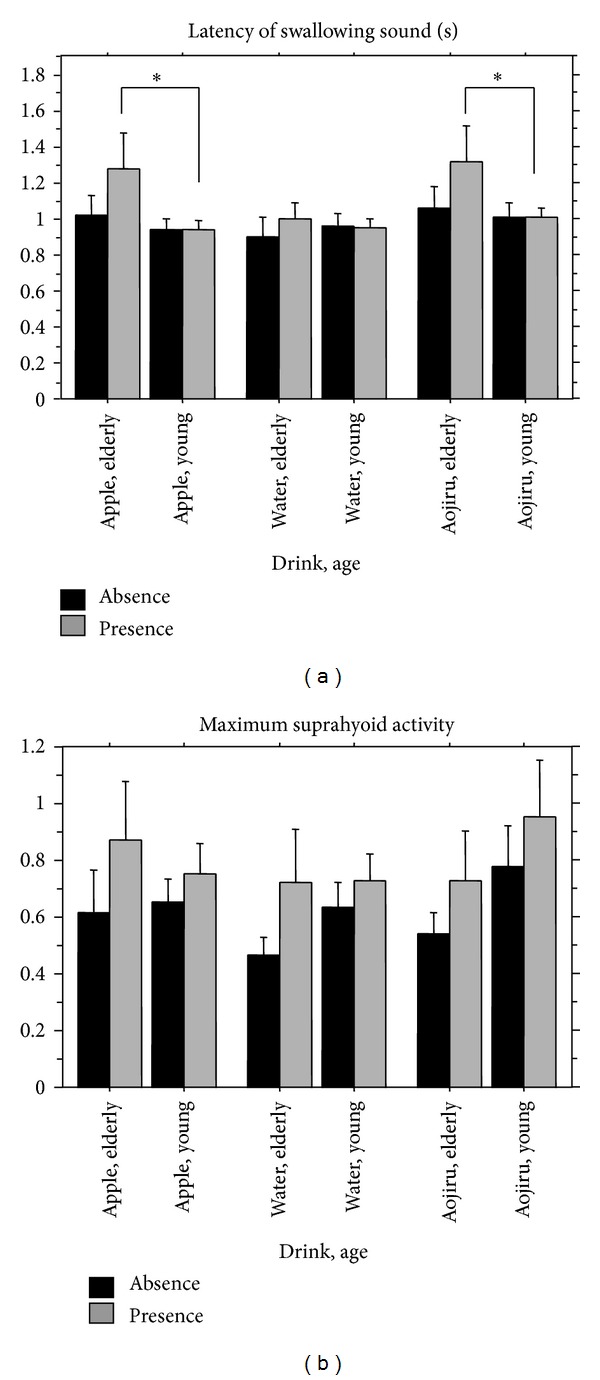
Comparisons of the latency of the swallowing sound and maximum suprahyoid muscle activity when swallowing with or without auditory verbal cues. Error bar: standard error.

*Latency of Swallowing Sound*. In the analysis of the latency of the swallowing sound, the ANOVA revealed a significant main effect of Age (*F*(1,330) = 5, *P* < .05), as shown in Figure 7(a). In the elder group, the latency of the swallowing sound was significantly longer compared to the younger group. There were no significant effects of Beverage or Cue on the latency of the swallowing sound.

*Maximum Suprahyoid Muscle Activity*. In the analysis of maximum suprahyoid muscle activity, the ANOVA revealed a significant main effect of Cue (*F*(1,330) = 5, *P* < .05), as shown in Figure 7(b). Maximum suprahyoid muscle activity significantly increased under the presence condition compared to the absence condition regardless of age. There were no effects of Beverage or Age on maximum suprahyoid muscles activity.

There were no significant effects of Beverage, Age, or Cue on the latency of suprahyoid muscle activity, the latency of infrahyoid muscle activity, maximum infrahyoid muscle activity, or the interval between the onset of the swallowing sound and the peak timing point of infrahyoid muscle activity. 

*Analysis 4*

**Figure 8:**
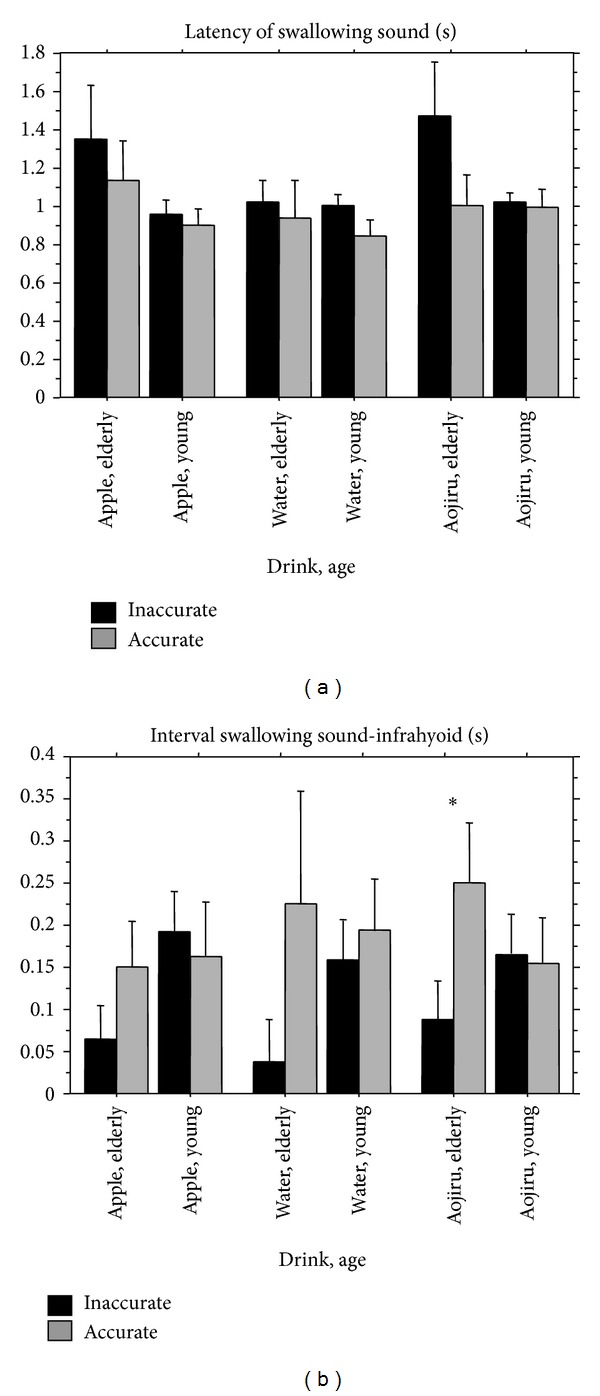
Comparisons of the latency of the swallowing sound and the interval between the onset of the swallowing sound and the peak timing of infrahyoid muscle activity during swallowing when the participant's anticipation was primed by auditory verbal cues that were accurate versus inaccurate. Error bar: standard error.

*Latency of Swallowing Sound*. In the analysis of the latency of the swallowing sound, the ANOVA revealed a significant main effect of Age (*F*(1,159) = 4.4, *P* < .05), as shown in Figure 8(a). In the elder group, the latency of the swallowing sound was significantly longer compared to the younger group. There were no effects of Beverage or Accuracy on the latency of the swallowing sound.

*Interval Swallowing Sound-Infrahyoid*. The ANOVA for the interval between the onset of the swallowing sound and the peak timing point of infrahyoid muscle activity revealed a significant main effect of Accuracy (*F*(1,159) = 4.2, *P* < .05), and a significant interaction effect between Accuracy and Age (*F*(2,159) = 4.3, *P* < .05), as shown in Figure 8(b). In the elder group, this interval was significantly shorter under the inaccurate condition than the accurate condition. In the younger group, there was no effect of accuracy on this interval. There were no effects of Beverage or Age on the length of the interval.

There were no effects of Beverage, Age, or Accuracy on the latency of suprahyoid muscle activity, the latency of infrahyoid muscle activity, maximum suprahyoid muscle activity, or maximum infrahyoid muscle activity. 

*Analysis 5*. The repeated-measures ANOVA revealed significant differences between the latency of the swallowing sound and muscle activity for both the younger group (*F*(2,143) = 30.2, *P* < .01) and the elder group (*F*(2,143) = 73.8, *P* < .01), as shown in Table 1. The latency of suprahyoid muscle activity was significantly shorter than that of the swallowing sound in the elder group (*P* < .01) but not in the younger group. The latency of the swallowing sound in the elder group was significantly longer than that in the younger group (*P* < .05).

## 4. Discussion

### 4.1. Characteristic Differences between the Beverages

The results from Experiment 1 are as follows. The taste score and swallowing ease score were the highest for apple and the lowest for aojiru. The distaste score was the lowest for apple and the highest for aojiru. Water was given an intermediate rating on these scales. These results indicate that the participants felt that apple juice has a good flavor and is easy to swallow, water has a neutral flavor and is easy to swallow, while aojiru has a bad flavor and is difficult to swallow.

### 4.2. Effect of Auditory Verbal Cues on Subjective Index

*Taste Score.* The results of Analysis 1 showed that Cue had a significant effect on taste score. The taste score significantly increased when beverages were ingested with auditory verbal cues. The results of analysis 2 showed that Accuracy had a significant effect on taste score. The taste score significantly increased when correct auditory verbal cues were given before the beverage was ingested. These results suggested that flavor perception increased when the participant's anticipation was primed by appropriate auditory verbal cues.

*Swallowing Ease Score*. The results of Analyses 1 and 2 showed a significant effect of auditory verbal cues on the swallowing ease score. The swallowing ease score significantly decreased when inaccurate auditory verbal cues were given before the beverages were ingested. These results suggest that ease of swallowing decreased when inappropriate anticipation was induced by an incorrect auditory verbal cue.

*Distaste Score*. Analyses 1 and 2 showed no significant effect of auditory verbal cues on distaste scores. Distaste plays a vital role in judgment about whether a potential food is edible. Earlier studies have reported that inedible foods are bitter and/or sour and connected with unpleasantness (distaste) [10]. Because this sensation is important for detecting potentially dangerous foods, it is logical that the perception of distaste is very sharp and stable. This may explain why auditory verbal cues had no effect on distaste score.

### 4.3. Effects of Auditory Verbal Cues on Physiological Index

Results obtained from analysis 3 showed that Cue had a significant effect on maximum suprahyoid muscle activity regardless of age. Maximum suprahyoid muscle activity significantly increased when beverages were ingested with auditory verbal cues. Previous studies have noted that the strongest activation of suprahyoid muscles is observed at the start of the pharyngeal reflex. Suprahyoid muscle activation is also observed before the start of the pharyngeal reflex when bolus is in the mouth [11]. 

The proper preparation for oral and/or pharyngeal swallowing, which was induced by the auditory verbal cues, might have been caused by an enhancement of suprahyoid muscle activity.

### 4.4. Effect of Age on Physiological Index

 It has been documented that healthy elderly individuals have slower overall tongue movements during the early portion of swallowing, a delay in triggering the pharyngeal component of swallowing, and an increased duration of the pharyngeal component of swallowing [7–9]. In this study, regardless of the presence or absence of a cue to ingest a beverage, the latency of the swallowing sound was significantly longer in the elder group than in the younger group. The swallowing sound is known to start when the pharyngeal reflex starts [11]. The results of this study showed that the pharyngeal reflex is more delayed in elderly individuals. These results are in accord with previous studies [7–9]. 

In contrast, this study showed no significant difference in the latency of suprahyoid muscle activity and the latency of infrahyoid muscle activity between the younger group and the elder group. Earlier studies have suggested that the suprahyoid muscles can be involved in both the oral and pharyngeal phases of swallowing (particularly in laryngeal elevation) [12–14]. 

Analysis 5 showed that in the younger group of participants the maximum suprahyoid muscle activity occurred concomitantly with the swallowing sound and occurred prior to the swallowing sound in the elder group. The activation of the suprahyoid muscles is known to be the strongest at the start of the pharyngeal reflex and is also observed during oral transit [11]. In agreement with previous research, the activation of the suprahyoid muscles was the strongest at the start of the pharyngeal reflex in the younger individuals. On the other hand, in elderly individuals, the activation of the suprahyoid muscles might be stronger during the oral transit of a beverage than during the start of the pharyngeal reflex because of a decline in tongue movement associated with age that does not impact younger individuals [15].

The elder group showed a significant delay in the swallowing sound, but not in the muscle activities, when auditory verbal cue was incorrect. The interval between the onset of the swallowing sound and the peak timing point of infrahyoid muscle activity was significantly shorter for incorrect auditory verbal cues than for correct ones in the elder group but not in the younger group. This shortening found only in the elderly may indicate that incorrect verbal cues may cause significant mismatch between the swallowing muscle control and actual beverage transition through pharynx and eventually might result in laryngeal penetration and/or aspiration. 

The hypofunction of swallowing [7–9, 15–18] and/or decreased taste and smell perception [19–24], with which elderly participants may suffer, may accelerate confusion in beverage recognition when inappropriate auditory verbal cues are given, thus altering swallowing behavior.

The present results suggest that effective verbal communication about food to be ingested is useful for improving flavor perception and for avoiding abnormal swallowing in young and elderly people. Individuals who require assistance with eating, such as those with dysphagia, as well as those with early dementia, attentional dysfunction, and disorders of consciousness may have difficulties recognizing appropriate times to eat and identifying food. Proper auditory verbal cues given with food to be ingested might be important in improving flavor perception and avoiding abnormal swallowing in these individuals.

## 5. Conclusion

Taste scores were significantly increased when a participant's anticipation was appropriately primed by a correct auditory verbal cue, while the swallowing ease score significantly decreased when the anticipation was contradicted. There was no significant effect of auditory verbal cue on the distaste score. Regardless of age, maximum suprahyoid muscle activity significantly decreased when beverages were ingested without auditory verbal cues. The interval between the onset of the swallowing sound and the peak timing point of infrahyoid muscle activity was significantly shortened when the anticipation induced by the auditory verbal cue was contradicted in the elderly participants. These results suggest that flavor perception and swallowing are affected by anticipation induced by auditory verbal cues. Disorders that affect the anticipatory stage of ingestion may cause subjective and physiological difficulties in swallowing; however, these difficulties can be abated with the use of adequate auditory verbal cues.

## Figures and Tables

**Table 1:** Comparisons of the latencies and swallowing sound for each of the muscles.

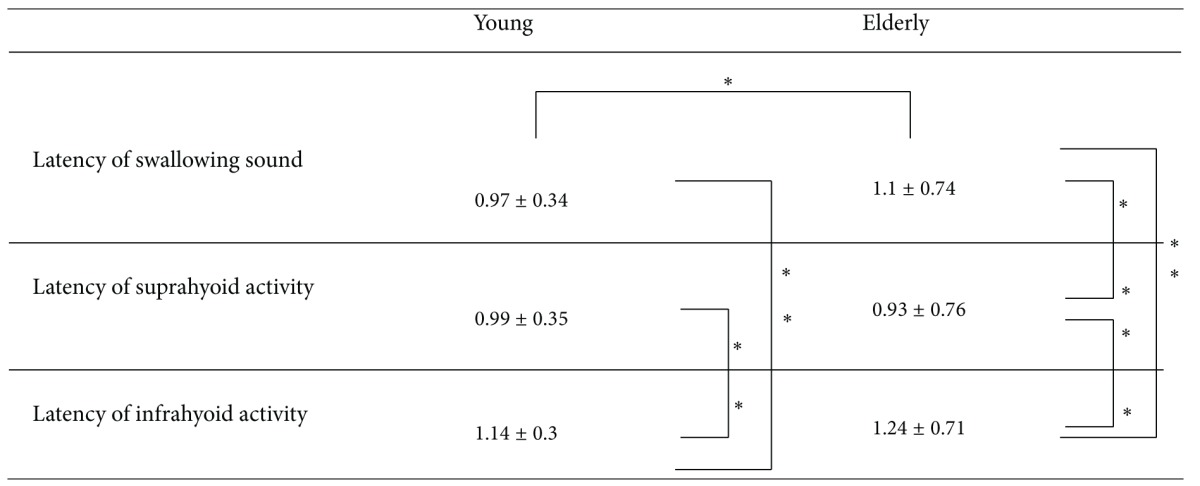

*P < .05, **P<.01.
